# Insights from the proteome profile of *Phytophthora capsici* in response to the novel fungicide SYP-14288

**DOI:** 10.7717/peerj.7626

**Published:** 2019-08-27

**Authors:** Meng Cai, Zhiwen Wang, Xiaoxia Ni, Yanhua Hou, Qin Peng, Xiang Gao, Xili Liu

**Affiliations:** 1 College of Chemistry, Central China Normal University, Wuhan, China; 2 Department of Plant Pathology, China Agricultural University, Beijing, China

**Keywords:** SYP-14288, *Phytophthora capsici*, iTRAQ, Mode of action, Proteome analysis

## Abstract

*Phytophthora capsica* is a destructive oomycete plant pathogen that causes huge losses to crop production worldwide. However, the novel fungicide SYP-14288 has shown excellent activity against various stages of the oomycete life cycle as well against fungal plant pathogens. The current study utilized isobaric tags for relative and absolute quantitation technology to generate proteome profiles of *P. capsici* in the presence or absence of SYP-14288 in order to gain a greater understanding of the SYP-14288 mode of action. A total of 1,443 individual proteins were identified during the investigation, of which 599 were considered to have significantly altered expression. Further investigation using Cluster of Orthologous Groups of proteins analysis and Kyoto Encyclopedia of Genes and Genomes pathway analysis indicated most of the proteins with altered expression were associated with carbohydrate metabolism, energy metabolism and their downstream biological functions, especially with regard to oxidoreductase activity and subsequent adenosine triphosphate (ATP) production associated pathways. Quantitative expression analysis using qRT-PCR validated the proteomic data. These results seem to indicate that SYP-14288 treatment caused a shift in energy metabolism that resulted in the activation of compensatory mechanisms affecting carbohydrate and lipid metabolism. The study also found evidence that the up-regulation of transmembrane transporters and proteins associated with stress response might also be coopted to compensate for the disrupted proton gradient and other downstream effects. Taken together these results provide strong evidence that SYP-14288 has a similar mode of action to the oxidative phosphorylation uncoupler Fluazinam but further investigation, including molecular studies, is required to completely characterize the SYP-14288 mode of action in *P. capsici*. However, the proteomic data collected in the current study does provide important insight into the overall effect of SYP-14288 in *P. capsici*, which could be useful for the registration and application of this novel fungicide.

## Introduction

The oomycete *Phytophthora capsici* Leonian is a destructive pathogen, which causes crown, root and fruit rot, in a wide range of solanaceous and cucurbit crops leading to significant economic losses throughout the world every year (Lamour et al., 2012). Indeed, its economic impact is so great that it is currently listed as one of the top 10 most important oomycete pathogens in plant pathology (Kamoun et al., 2015). Although genetic modification of susceptible species and preventative management practices have improved the control of diseases caused by *Phytophthora capsici*, the application of fungicides is still a critical component of integrated control programs (Ristaino & Johnston, 1999).

The novel fungicide SYP-14288, which was developed by Shenyang Research Institute of Chemical Industry of China, belongs to the diarylamine family, and shares a similar chemical structure with Fluazinamn (Fig. S1), which is reported to be an uncoupler of mitochondrial oxidative phosphorylation (Guo et al., 1991). Like other diarylamine fungicides, SYP-14288 exhibits broad-spectrum fungicidal activity that provides excellent control of many plant diseases including *Colletotrichum orbiculare* and cucumber downy mildew *Pseudoperonospora cubensis*, as well as outstanding control of the pepper blight caused by *Phytophthora capsici*. Although SYP-14288 works as an uncoupler to control various plant pathogens, its exact target site(s) remains unclear (Wang et al., 2018). Given the great potential of SYP-14288 within the fungicide market, it is important to characterize its mode of action in greater detail.

The completion of the *Phytophthora capsici* genome sequencing project has provided the opportunity for new analytical techniques to be applied to the study of fungicide mode of action in this important pathogen including proteomics, which has been widely applied for the toxicological evaluation of novel chemicals, and especially target site discovery in recent years (Bruneau et al., 2003; Hughes et al., 2006; Bantscheff et al., 2007). For example, two-dimensional gel electrophoresis has been successfully used to investigate the response of *Phytophthora cactorum* to zoxamide (Mei et al., 2014), while MALDI-TOF-MS/MS has been used to explore the mode of action of the novel fungicide JS399-19 in *Fusarium graminearum* (Hou et al., 2013). Similarly, the isobaric tags for relative and absolute quantitation (iTRAQ) method has been used to study the effect of pyrimorph in *Phytophthora capsici* (Pang et al., 2015). Proteomic methods provide important information that is difficult to obtain using traditional biochemical studies and have therefore become popular tools to study the mode of action of novel compounds.

The current study utilized a quantitative proteomics approach based on iTRAQ technology to investigate the changes in protein expression that occur in *Phytophthora capsici* exposed to SYP-14288 in order to gain a global perspective of the biochemical pathways affected by SYP-14288, as well as to identify specific target sites that could be validated by qRT-PCR. It is hoped that the results of the study will provide an expanded understanding of the SYP-14288 mode of action as well as critical data that can be utilized for the design of novel fungicides against a broad range of plant diseases.

## Materials and Methods

### Sensitivity of *Phytophthora capsici* to SYP-14288

Mycelial growth inhibition method as previously described in Cai et al. (2016) was used to determine the sensitivity of *Phytophthora capsici* in response to treatment with SYP-14288. Six *Phytophthora capsici* isolates collected from peppers of different breeding fields in China were evaluated. All the isolates were cultured in darkness on potato dextrose agar plates (200 g potato, 20 g Glucose, 15 g agar, distilled water to one L) containing SYP-14288 (kindly provided by Shenyang chemical research institute) at the following concentrations: 0, 0.3, 0.6, 1, 5, 10, 50 µg/mL, respectively. Dimethyl sulfoxide was used as a solvent control. The plates were then incubated at 25 °C for 3 days before the diameter of each colony was measured at two perpendicular angles. Each treatment consisted of four replicate plates and the entire experiment conducted three times. The half maximal effective concentration (EC_50_) was then calculated using a regression equation (Supplementary Information) derived by correlating the log of the SYP-14288 concentration and the probit of percentage inhibition based on the average radial growth of the *Phytophthora capsici* isolates compared to the control treatment.

### Protein extraction from *Phytophthora capsici*

According to the previously described method in Pang et al. (2015), three g of mycelial samples harvested from the wild type isolate HD3 were incubated in 80 mL PDB medium amended with one µg/mL SYP-14288 (the EC_50_ determined above). Equivalent control samples were prepared in the absence of SYP-14288. The liquid cultures were then placed in dark-incubation at 25 °C for 24 h in a shaker (220 rpm) before the mycelia were harvested by centrifugation. The resulting mycelia were washed and dried under vacuum before being stored at −80 °C until required. A total of 10 separate cultures were used for each treatment and the entire experiment conducted once.

### Preparation of protein hydrolysate

The frozen mycelia from each treatment were ground under liquid nitrogen in a ball mill (MM400; Retsch, Haan, Germany) using two mL tubes and five mm sterile steal balls, before two volumes of cold (4 °C) lysis buffer (8 M urea, 30 mM HEPES, one mM PMSF, two mM EDTA, and 10 mM dithiothreitol (DTT)) were added to each tube. The samples were then subjected to low temperature sonication (Sonics Vibra-Cell VCX-500 Ultrasonic Processor; Artisan, Champaign, IL, USA) (pulse on 2 s, pulse off 3 s, power 180 W) for 5 min to enhance cell lysis, before centrifugation at 20,000×*g* for 30 min. The supernatant containing the protein fraction was then transferred to new tubes, while the pellet containing undisrupted cells was discarded. DTT was added to each sample to a final concentration of 10 mM before incubation at 56 °C. After 1 h iodoacetamide was added to a final concentration of 55 mM before a second period of dark-incubation (1 h). The protein was then precipitated by the addition of four volumes of cold acetone buffer and incubation at −20 °C for 3 h before being collected by centrifugation at 20,000×*g* for 20 min at 4 °C. The resulting protein samples were then washed in cold acetone to remove any residual DTT before being dissolved in 500 µL resuspension buffer (50% w/v tetraethylammonium bromide, 0.1% SDS). The concentration of the protein samples was measured using the Bradford dye-binding assay (Bradford, 1976), and an equal amount of protein from each of the 10 replicates pooled together to produces 100 µg samples for each treatment. The resulting samples were then hydrolyzed by one µg/µL trypsin at 37 °C overnight and dried in vacuum before being analyzed.

### iTRAQ labeling

The peptides from the two treatments (SYP-14288 and negative control) were labeled using the iTRAQ Reagent-8 Plex Multiplex kit (Applied Biosystems, Waltham, MA, USA) according to the protocol of the manufacturer. Different iTRAQ tags were used for the two treatments. The labeled peptides were then dried under a speed-vac before being assessed by chromatography.

### Segregation of peptides by chromatography

The peptide mixtures were separated according to the reported protocol in Pang et al. (2015). The samples were first dissolved in 100 µL buffer A (10 mM KH_2_PO_4_, pH 3.0; acetonitrile ACN/H_2_O 25/75, v/v) before flowing through a 100 × 4.6 mm fractionation column (Luna^®^; Phenomenex, Inc., Torrance, CA, USA). The proteins were then eluted using a gradient of two buffers: 100% buffer A for 10 min, 0–30% buffer B (10 mM KH_2_PO_4_, pH 3.0; 2 M KCl, ACN/H_2_O 25/75 v/v) for 15 min, 30–100% buffer B for 15 min and 100% buffer B for 10 min at a flow rate of one mL/min for 1 h. The UV absorbance of the eluents was detected at 214 nm. A total of 16 fractions were acquired and dried before being desalted using a C18 column (Strata-X; Phenomenex, Inc., Torrance, CA, USA).

### Analysis of peptides by LC–MS/MS

Peptide fractions were analyzed according to the previously described proteomics study in Pang et al. (2015). In brief, the LC–MS/MS system consisting of the Thermo Fisher Scientific Easy-nLC nanoflow chromatograph and the Thermo Fisher Scientific Q Exactive™ Mass Spectrometer was used. The samples were fractionated in a 100 × 75 mm C18 column (PepMap™; Thermo Fisher Scientific, Waltham, MA, USA) at a flow rate of 400 nL/min. The MS spectra in the range of 350–2,000 m/z were acquired in the positive ion reflection mode using a data-dependent Top 10 method which dynamically selected the most abundant precursor ions from the survey scan (300–1,650 Th) for higher energy collisional dissociation fragmentation (Michalski et al., 2011).

### Database searches for peptide and protein identification

The raw MS/MS data were converted into the .MGF file format using the Proteome Discoverer 1.3 software (Thermo Fisher Scientific, Waltham, MA, USA), before the exported files were used to search the NCBI *Phytophthora* Database employing the automatic decoy database search function of Mascot 2.3 (Matrix Science, London, UK). Several parameters in Mascot were optimized for peptide searching, including iTRAQ 8-plex for quantification, tolerance of one missed cleavage of trypsin, carbamidomethylation for cysteine as a fixed modification, and oxidation for methionine as a variable modification. The precursor mass tolerance was set to 15 ppm and the mass tolerance to 20 mmu. A concatenated target-decoy database-search strategy was used to check the rate of false positives, which were found to be less than 1% in all cases. High-confidence proteins which contained at least two unique peptides with a protein score greater than 1.3 were selected for further quantification.

### Quantitative data analysis of iTRAQ labeled peptides

The iTRAQ quantification was determined based on the reporter ion intensities detected from Proteome Discoverer version 1.3 (Thermo Fisher Scientific, Waltham, MA, USA). The iTRAQ channels were normalized by summing all the reporter ion intensities in each iTRAQ channel and equalizing each channel contribution by dividing individual reporter ion intensities by the corresponding channel-specific correction factor. The quantitative data was analyzed using statistical procedures previously described in Ross et al. (2004), where the log2-transformation for the iTRAQ ratios was first performed to yield a normal distribution. Proteins were considered to be differentially expressed if they displayed at least 1.5-fold change in abundance (±) in response to SYP-14288 treatment, and therefore were selected as proteins of interest in the subsequent Kyoto Encyclopedia of Genes and Genomes pathway (KEGG) analysis. The KEGG analysis was performed according to the formula described previously (Liu et al., 2012), and the pathways with a *P*-value <0.05 were considered significantly “enriched” to be correlated with the altered expression of proteins in response to SYP-14288. A Cluster of Orthologous Groups of proteins (COG) analysis was further performed according to the previously described method in Ye et al. (2006).

### Total RNA extraction and qRT-PCR validation of differentially expressed proteins

The qRT-PCR analysis of selected *Phytophthora capsici* genes belonging to different KEGG pathways were performed using Total RNA extracted from mycelial samples prepared according to the protocol described above regarding protein samples. The RNA was extracted using a TRIzol method, and first strand cDNA synthesized using the TransScript™ Two-Step RT-PCR SuperMix kit (Transgen, Beijing, China). The qRT-PCR analysis was performed using SYBR Green II fluorescent dye and the ABI PRISM 7500 Real-Time PCR system (Applied BioSystems, Foster City, CA, USA). The 20 µL reaction mixtures contained 10 µL of SYBR Premix DimeEraser (2×), 0.4 µL of ROX Reference Dye Dye II (50×conc.), two µL of template DNA, 0.6 µL of each of the forward and reverse primers (10 µM), and 6.4 µL of ddH_2_O, and processed according to the following program: an initial melting at 95 °C for 30 s; followed by 40 cycles of 95 °C for 5 s, 57.3 °C for 30 s, and 72 °C for 34 s, with a final extension at 95 °C for 30 s. The melting curves were obtained using a standard protocol, and used to confirm that the target products did not contain primer dimers after the amplification had been completed. Two housekeeping genes, WS21 and UBC, were selected as the internal controls, and their geometric mean Ct values used to determine the uniformity of the cDNA concentrations (Yan & Liou, 2006). Relative expression values were then calculated according to the 2^−ΔΔCT^ method (Livak & Schmittgen, 2001). Three biological replicates were performed for each of the two treatments using fresh cultures for the RNA extraction, while three technical replicates were performed for each biological replicate during the qRT-PCR itself. Statistical analysis for the qPCR data (*P* = 0.05) was carried out as described previously (Yuan et al., 2006). The gene-specific primers used during the qRT-PCR analysis are listed in Table S1.

## Results

### Inhibitory effect of SYP-14288 on the mycelial growth of *Phytophthora capsici*

**Figure 1:**
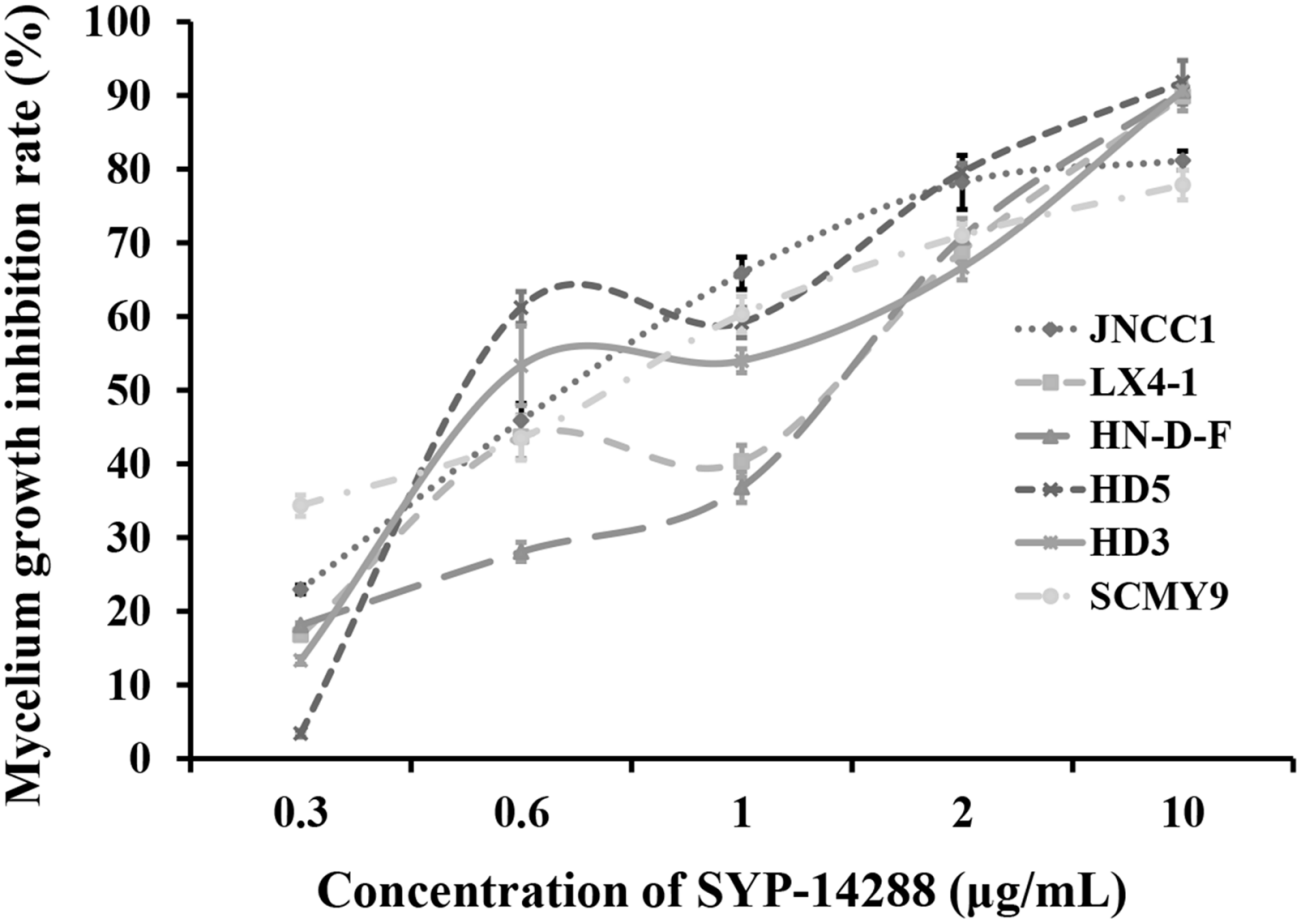
Dose-response curves illustrating the mycelial growth inhibition of six *P. capsici* isolates resulting from treatment with the novel fungicide SYP-14288.

Treatment with SYP-14288 was found to inhibit the mycelial growth of all the test isolates in a dose-dependent manner (Fig. 1). The EC_50_ of SYP-14288 varied, but was found to be approximately one µg/mL in isolate HD3, the isolate subsequently selected for the proteomic analysis. Previous studies have shown that a reduction of 50–70% during the exponential growth phase is ideal for proteomic analysis of fungicide effects (Bandow et al., 2003; Mei et al., 2014), while prolonged exposure can induce non-specific effects such as cell lysis, which can complicate the interpretation of proteomic data. Consequently the proteomic analysis was performed on samples collected from isolate HD3 after 24 h exposure to one µg/mL SYP-14288 (Mei et al., 2014; Pang et al., 2015).

### Overview of the quantitative proteomics analysis

A total of 1,443 proteins were identified during the iTRAQ–LC–MS/MS analysis of the two proteome libraries (Table S2). The majority of the proteins had a molecular mass in excess of 10 kDa, although the mass of individual proteins varied greatly, ranging from 5.5 to 726.6 kDa (Fig. S2).

### Changes in functional pathways in response to SYP-14288

**Figure 2:**
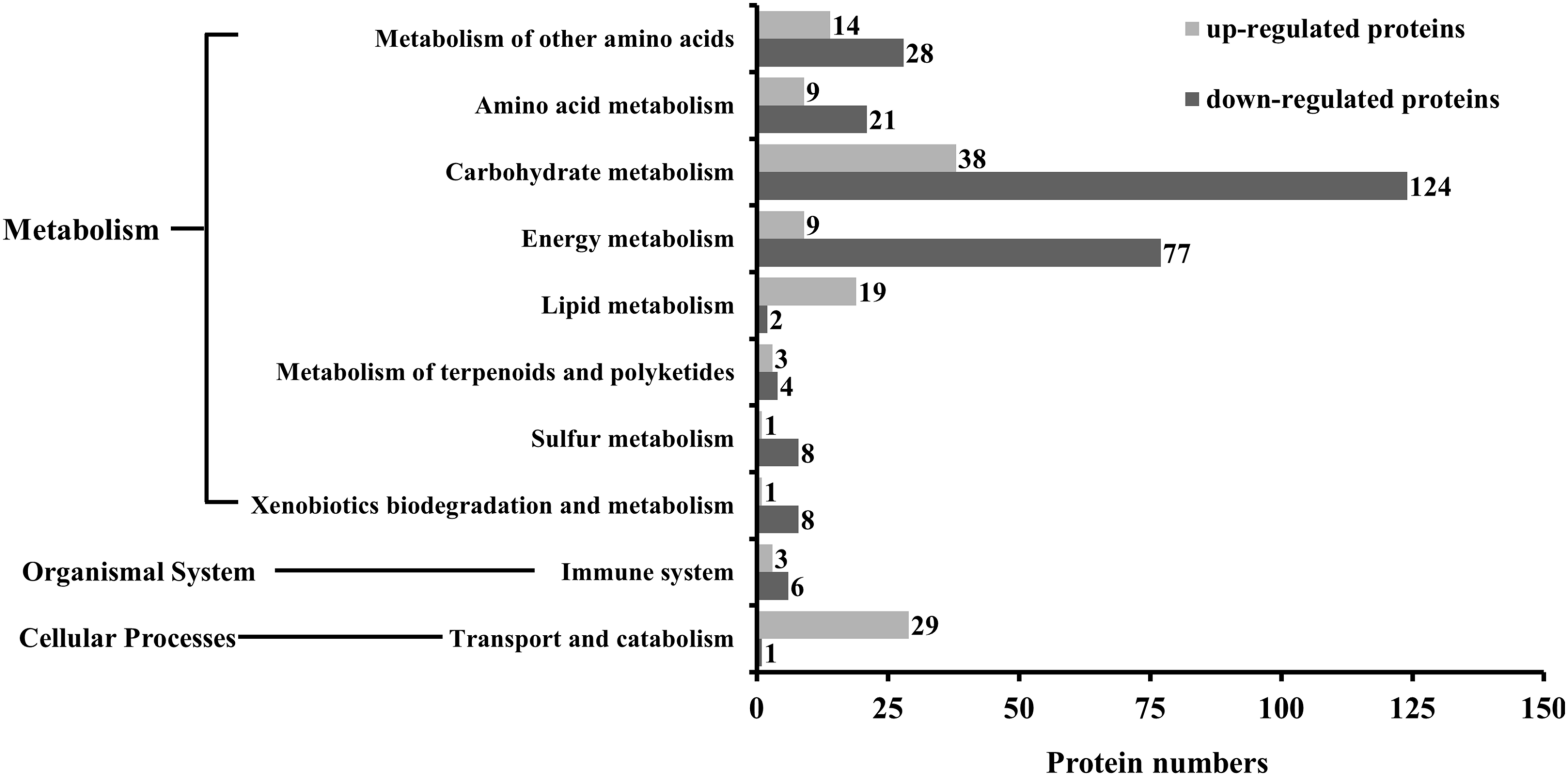
The number of differentially expressed proteins associated with each functional category during the KEGG analysis of *P. capsici* exposed to the novel fungicide SYP-14288.

The KEGG analysis indicated that as many as 186 pathways were affected by SYP-14288 treatment, including six primary pathways and 39 secondary pathways. However, only 18 specific pathways belonging to 10 different secondary pathways and three primary pathways were found to be significant (*P* < 0.05). These pathways included selenoamino acid metabolism, fatty acid metabolism, and the citrate cycle, and mostly belonged to the metabolism category, with only one pathway being allocated to the cellular processes category, and one to the organismal systems category (Table 1). The KEGG analysis indicated that the 10 secondary pathways affected by SYP-14288 were associated with the differential expression of 405 proteins, 126 of which were up-regulated and 279 of which were down-regulated. However, the majority of the proteins affected belonged to just five secondary pathways including carbohydrate metabolism, energy metabolism, metabolism of other amino acids, amino acid metabolism, transport and catabolism, which were associated with 162, 86, 42, 30 and 30 proteins, respectively (Fig. 2).

**Table 1:** KEGG pathways significantly altered in *P. capsici* exposed to the novel fungicide SYP-14288 (*P* < 0.05).

Pathway	*P*-value	Pathway ID	Functional category	
			Secondary category	Primary category
Selenoamino acid metabolism	0.003	ko00450	Metabolism of other amino acids	Metabolism
Fatty acid metabolism	0.005	ko00071	Lipid metabolism	Metabolism
Citrate cycle (TCA cycle)	0.006	ko00020	Carbohydrate metabolism	Metabolism
NOD-like receptor signaling pathway	0.010	ko04621	Immune system	Organismal systems
Pyruvate metabolism	0.013	ko00620	Carbohydrate metabolism	Metabolism
Carbon fixation in prokaryotes	0.013	ko00720	Energy metabolism	Metabolism
Beta-alanine metabolism	0.013	ko00410	Metabolism of other amino acids	Metabolism
Propanoate metabolism	0.016	ko00640	Carbohydrate metabolism	Metabolism
Cysteine and methionine metabolism	0.019	ko00270	Amino acid metabolism	Metabolism
Histidine metabolism	0.020	ko00340	Amino acid metabolism	Metabolism
Lysosome	0.021	ko04142	Transport and catabolism	Cellular processes
Methane metabolism	0.021	ko00680	Energy metabolism	Metabolism
Taurine and hypotaurine metabolism	0.028	ko00430	Metabolism of other amino acids	Metabolism
Limonene and pinene degradation	0.028	ko00903	Metabolism of terpenoids and polyketides	Metabolism
Sulfur metabolism	0.031	ko00920	Sulfur metabolism	Metabolism
Carbon fixation in photosynthetic organisms	0.035	ko00710	Energy metabolism	Metabolism
Glycolysis/Gluconeogenesis	0.042	ko00010	Carbohydrate metabolism	Metabolism
Chloroalkane and chloroalkene degradation	0.047	ko00625	Xenobiotics biodegradation and metabolism	Metabolism

### Changes in the expression of individual proteins in response to SYP-14288

A total of 599 individual proteins were found to have altered expression in response to SYP-14288, when a 1.5-fold change in abundance was used as a threshold for differential expression. Of these, 291 were found to be up-regulated, while 308 were down-regulated. Furthermore, 40 unique proteins were identified that were only expressed in *Phytophthora capsici* isolate HD3 in the presence of SYP-14288.

**Figure 3:**
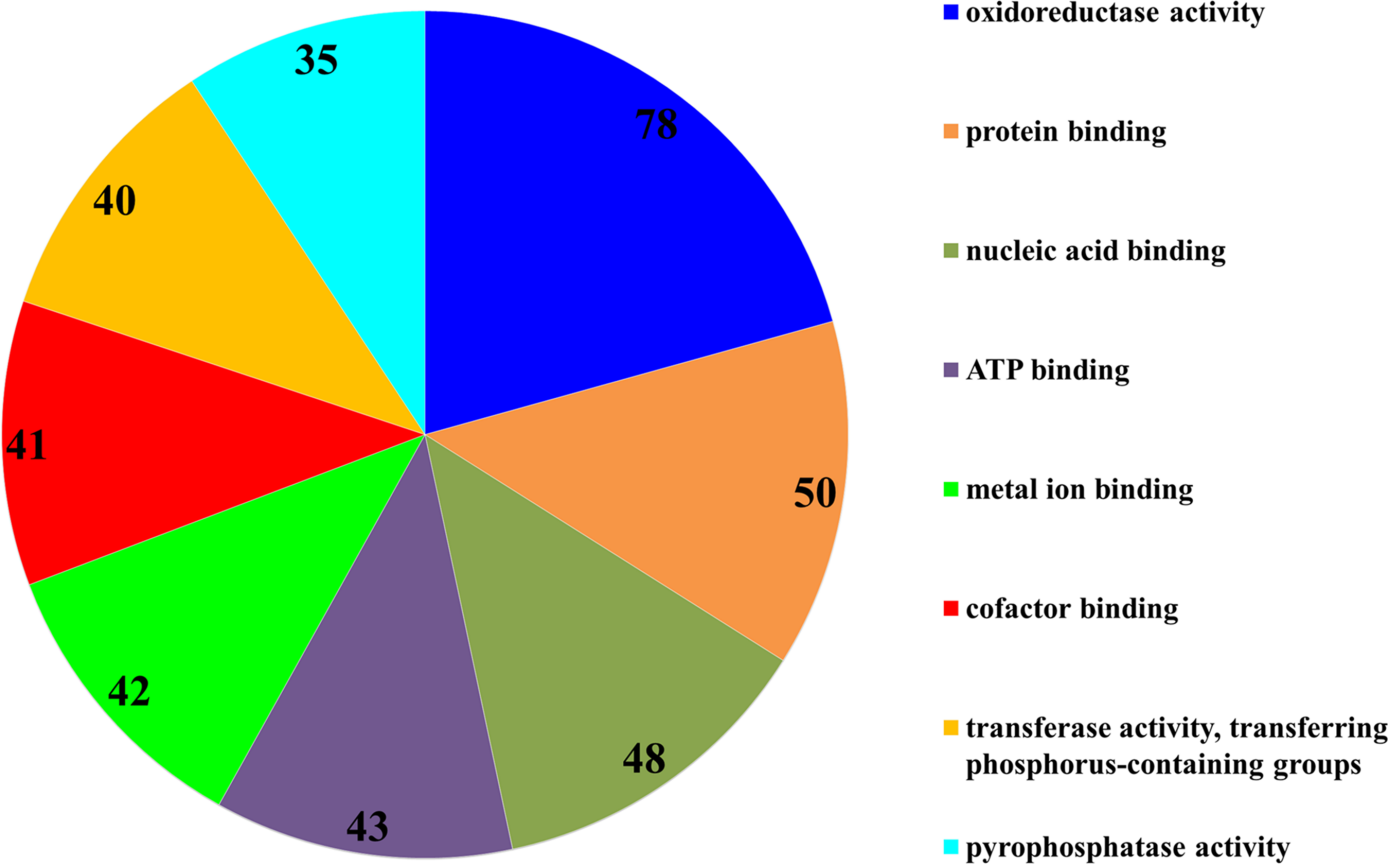
Functional categorization of proteins with most significantly altered expression in *P. capsici* exposed to SYP-14288 according to COG analysis. The numbers in each segment represent the number of proteins allocated to that functional category.

**Figure 4:**
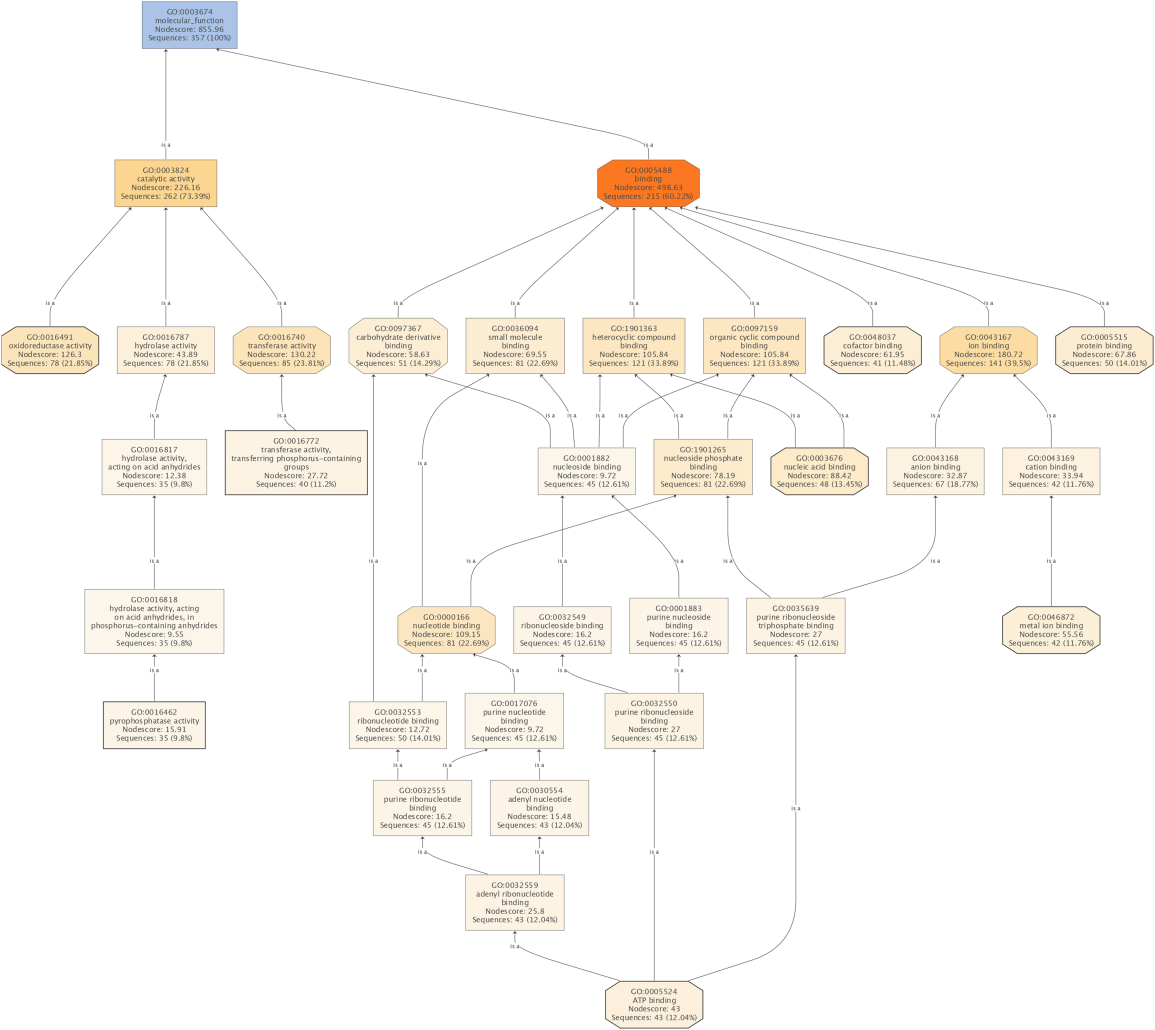
The molecular functions affected by SYP-14288 treatment of *P. capsici*. The ranking, top to bottom indicates relationships between the different functional categories, from primary effects to subordinate ones.

The subsequent COG analysis revealed that the proteins exhibiting differential expression were primarily associated with eight functional groups reflecting different molecular functions (Fig. 3). The oxidoreductase activity category contained the highest number of proteins, 78 in total, which was almost double in comparison to the other categories including protein binding, nucleic acid binding, ATP binding, metal ion binding, cofactor binding, transferase activity (transferring phosphorus-containing groups) and pyrophosphatase activity, which contained 50, 48, 43, 42, 41, 40 and 35 proteins, respectively. The COG analysis also indicated that most of the proteins affected by SYP-14288 exposure belonged to five primary functional categories including ATP binding, pyrophosphatase activity, oxidoreductase activity, transferase activity (transferring phosphorus-containing groups) and metal ion binding which mediated the changes occurring in a multitude of downstream pathways, thus, these should be the original pathways of the effect of SYP-14288 application. However, it should be noted that individual proteins can be assigned to more than one category during COG analysis (Fig. 4).

### *Phytophthora capsici* proteins associated with the SYP-14288 mode of action and stress response

Given that SYP-14288 has a similar chemical structure to the oxidative phosphorylation uncoupler Fluazinam, and likely shares the same mode of action, the results of the metabolic pathway enrichment analysis, which assigns metabolic function of differentially expressed proteins by matching them to previously annotated proteins in the KEGG pathway database, was used to investigate proton producing and transporting proteins and those associated with generalized stress responses (Table 2). The analysis revealed that many of the proteins associated with oxidative phosphorylation had altered expression in response to SYP-14288 were associated with proton production as well as proton-motive force formation and ultimately impacted the production of ATP. Other proteins associated with proton translocation and supplemental energy production, including ATP-binding Cassette (ABC) transporters and fatty acid metabolism were also affected. Similarly the heat shock proteins associated with energy release to protect organisms under stress were also found to be affected. These results provide strong evidence that SYP-14288 affects the energy metabolism of *Phytophthora capsici* and has a mode of action similar to Fluazinam.

**Table 2:** Differentially expressed proteins associated with the SYP-14288 mode of action and stress response in *P. capsici*.

Pathway	GenBank accession number	Protein description	Expression level^a^
Oxidative phosphorylation	262107486	Conserved hypothetical protein	0.61
	145932435	Cytochrome c oxidase subunit 3	0.637
	325187848	Conserved hypothetical protein	0.644
	262102866	NADH dehydrogenase flavoprotein 2	0.657
	348666689	Hypothetical protein PHYSODRAFT_566176	0.659
	145932440	NADH dehydrogenase subunit 7	1.505
	348670330	ATP synthase beta subunit	1.524
	262097555	Conserved hypothetical protein	1.577
	348667962	Hypothetical protein PHYSODRAFT_288955	1.614
	262095673	Hypothetical protein PITG_19772	1.617
	262111983	ATP synthase subunit beta	1.633
	262100029	Plasma membrane H+-ATPase	1.757
	348681621	Hypothetical protein PHYSODRAFT_285609	1.769
	348679595	Proton pump, proton transport	1.852
	348671348	Hypothetical protein PHYSODRAFT_287246	1.915
	348673808	Hypothetical protein PHYSODRAFT_355001	2.097
	407079205	Cytochrome c oxidase subunit 2, partial	2.123
	348677015	Hypothetical protein PHYSODRAFT_351065	2.157
	262104542	Vacuolar proton translocating ATPase A subunit	6.334
ABC transporters	262096576	ATP-binding Cassette (ABC) Superfamily	1.501
	348670516	pdr transporter	1.707
	262095970	ATP-binding Cassette (ABC) Superfamily	2.176
Carbohydrate metabolism	262108121	Pyruvate, phosphate dikinase	0.174
	262107575	Glycoside hydrolase, putative	2.042
Fatty acid metabolism	262105742	acyl-CoA dehydrogenase	2.048
	262106971	acyl-CoA dehydrogenase	3.619
Antigen processing and presentation	262099349	Heat shock protein 70	1.69
NOD-like receptor signaling pathway	262111229	Heat shock protein 90	1.576
RNA degradation	262106488	Heat shock 70 kDa protein, mitochondrial precursor	0.566
Uncharacterized	262108152	ATPase	1.957
	262105670	Inorganic phosphate transporter, putative	2.084

**Note:**

a The fold change value calculated by dividing expression levels in the SYP-14288 treatment by those of the control. Values greater than one indicate up-regulation, while those less than one indicate down-regulation.

### Gene expression analyses for differentially expressed proteins

**Figure 5:**
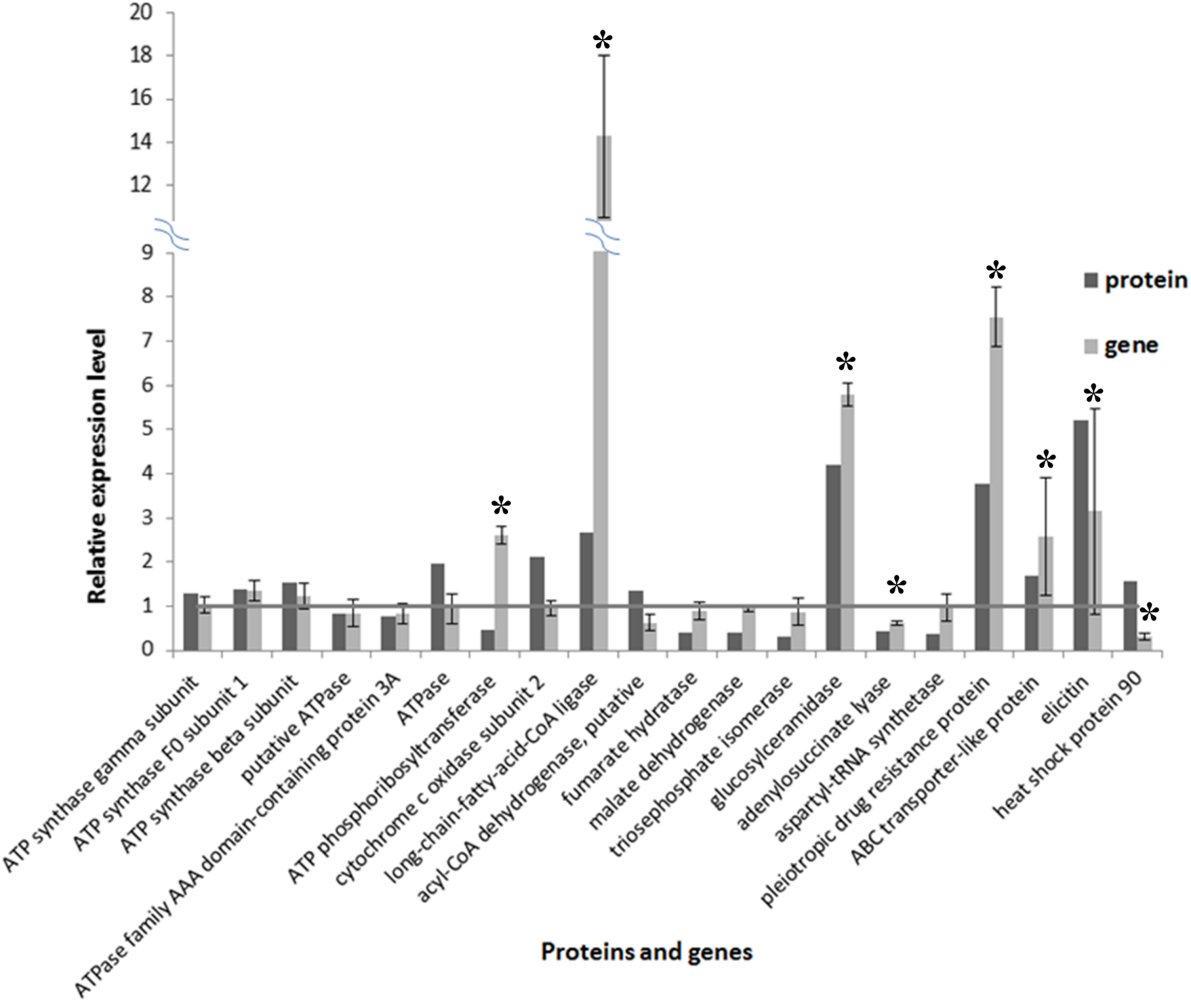
Comparison of protein and RNA expression levels for 20 *P. capsici* genes/proteins with altered expression in response to the novel fungicide SYP-14288. An * (asterisk) indicates the gene shows significantly differential expression.

A total of 20 proteins belonging to different metabolic pathways associated with the effect of SYP-14288 were selected to validate the results of the proteomic analysis using qRT-PCR. The results confirmed that 16 of the 20 genes had altered trends of expression consistent with the proteomic data, which implied the proteomic data was reliable (Fig. 5). According to the statistical analysis, eight of them differed significantly (*P* < 0.05). Besides, four genes including ATPase, ATP phosphoribosyltransferase, heat shock protein 90 and a putative acyl-CoA dehydrogenase had different altered trends with proteomic data, but the difference were not huge (with protein/gene levels at 1.957/0.941, 0.475/2.602, 1.576/0.297 and 1.341/0.621, respectively).

## Discussion

Proteomic analysis is a powerful tool to determine the mode of action of fungicides by identifying proteins and metabolic pathways that have altered patterns of expression in response to exogenous compounds (Vido et al., 2001; Teixeira et al., 2005; Bantscheff et al., 2007; Santos, Simoes & Sa-Correia, 2009). The current study employed an iTRAQ LC–MS/MS based approach to produce quantitative proteome profiles to characterize the response of *Phytophthora capsici* to the novel fungicide SYP-14288. A total of 1,443 proteins were identified in the control and EC_50_ treatment libraries. The subsequent gene ontology analysis categorized these proteins into 186 biological processes, 18 of which had significantly altered expression in the fungicide treatment, including metabolic pathways such as carbohydrate and amino acid metabolism. Although only 18 pathways were affected, a total of 599 individual proteins were found to have significantly altered expression (change fold ≥ 1.5), the majority of which were associated with carbohydrate and energy metabolism. The subsequent COG analysis categorized the proteins into eight groups including oxidoreductase activity, protein binding and nucleic acid binding. Several of the proteins identified by the COG and KEGG pathway analysis were selected for validation using qRT-PCR, which primarily focused on proteins associated with oxidative phosphorylation, ATP consumption, proton transportation and responses to stress that would all be affected if SYP-14288 had a similar mode of action as the fungicide Fluazinam. For the most part, the results of the qRT-PCR were consistent with those of the iTRAQ analysis, with most of the genes having similar patterns of expression, which indicated that the proteomic analysis was convincing and reliable. However, there were a few disparities between the proteome and RNA data, which most like resulted from a time lag between transcription and the accumulation of the resulting protein.

Many of the proteins (86 in total) that had altered expression in response to SYP-14288 were associated with energy metabolism, with many being significantly up-regulated. For example, the ATP synthase subunit beta, which is a component of the ATP synthase complex that catalyzes ATP synthesis by utilizing the electrochemical gradient of protons across the inner membrane of the mitochondrion produced during oxidative phosphorylation (Wang & Oster, 1998; Elston, Wang & Oster, 1998; Tyler et al., 2006; Leyva, Bianchet & Amzel, 2009). The vacuolar proton translocating ATPase A subunit and an uncharacterized ATPase (GI: 262108152) were also found to be highly up-regulated. It is well established that ATPase enzymes can function as pumps (including proton pumps) and as co-transporters, catalyzing ATP hydrolysis during the process to provide metabolic energy (Martin & Senior, 1980; Njus, Knoth & Zallakian, 1981). Previous research has shown that the up-regulation of these proteins and the supplemental energy produced by their activity can play an important role in drug resistance and during severe oxidative stress (Milgrom et al., 2007). The up-regulation of these two proteins appears to corroborate the hypothesis that SYP-14288 acts as an uncoupler of oxidative phosphorylation or as an ATP biosynthesis inhibitor similar to Fluazinam. The current study also found that several proteins associated with the oxidative phosphorylation pathway itself were significantly down-regulated, including cytochrome c oxidase subunit 3 (also known as complex IV of respiratory electron transport chain) and NADH dehydrogenase flavoprotein 2, which are both involved in proton transfer and the establishment of the transmembrane proton gradient in mitochondria that provides the electrochemical potential for the ATP synthase (Hochstein & Dalton, 1973; Tsukihara et al., 1995; Balsa et al., 2012). The down-regulation of these proteins suggests that SYP-14288 may cause a high proton gradient between the inner membrane of the mitochondrion that can inhibit the formation of proton pumps on the outer surface of the inner membrane via a negative feedback mechanism. In addition, it was interesting to note that the NADH dehydrogenase subunit 7 was significantly up-regulated (change fold = 1.505) in response to SYP-14288. This protein, which is one of the main sources of superoxide and is responsible for the translocation four protons across the mitochondrial membrane, has previously been reported to be the primary target of the uncoupler Fluazinam (Lee et al., 2012), which can be taken as further evidence that the two fungicides have shared a mode of action.

Many of the proteins with altered expression were found to be involved in carbohydrate metabolism. The most down-regulated protein in this category was pyruvate phosphate dikinase, which is responsible for phosphoenolpyruvate biosynthesis, a process that requires ATP (Evans & Wood, 1968; Pocalyko et al., 1990; Mei et al., 2014), the down regulation of which could have been caused be a reduction in ATP as a result of SYP-14288 treatment. In contrast, several glycolytic enzymes, including glycoside hydrolases that assists in the hydrolysis of glycosidic bonds in complex sugars (Sinnott, 1990; Davidson et al., 2006), were up-regulated. For example, glyceraldehyde-3-phosphate dehydrogenase, which catalyzes the sixth step in glycolysis (Tarze et al., 2007), was highly up-regulated (change fold = 2.275). The increased expression of enzymes associated with sugar metabolism enzymes could be another response to increase energy generation to compensate for the disrupted proton gradient caused by the putative uncoupling effect of SYP-14288. The activation of glucose consumption is not only a means to produce essential energy but has also been associated with mechanisms that respond to metabolic stress (Owen et al., 2008). The results of the current study also indicated that many downstream proteins associated with the processing of acetyl-CoA in citric acid cycle, pyruvic acid metabolism and the pentose phosphate pathway were down-regulated. It is possible that these changes occurred in response to reduced acetyl-CoA levels, which could imply that SYP-14288 has an effect on enzymes that catalyze the conversion of pyruvic acid to acetyl-CoA increasing the production of ATP via the citric acid cycle, another mechanism that could compensate for the reduced levels of ATP caused by uncoupler activity.

Aside from changes that directly affected energy metabolism, the current study also found that SYP-14288 changed the expression of proteins involved in amino acid and lipid metabolism including aminotransferase, which catalyzes the interconversion of certain amino acids and α-keto acid (Grenville-Briggs et al., 2005); argininosuccinate lyase (change fold = 0.521), which is involved in arginine biosynthesis (Knudsen, Burton & Færgeman, 2003); translation elongation factor 1 alpha, which is involved in polypeptide elongation (Van’t Klooster et al., 2000); and the hypothetical protein PHYSODRAFT-485399; which all had reduced levels of expression. The negative impact of SYP-14288 on such a wide range of proteins associated with amino acid metabolism indicates that it has a significant effect on protein synthesis in *Phytophthora capsici*. Similar results have also been observed in the response of *Phytophthora capsici* to Pyrimorph (Pang et al., 2015). In contrast, almost all of the proteins involved in lipid metabolism were up-regulated including Acyl-CoA dehydrogenase, which catalyzes the initial step in each cycle of fatty acid β-oxidation resulting in the introduction of a trans double-bond between C2 and C3 of the acyl-CoA thioester substrate (Thorpe & Kim, 1995); and a member of the enoyl-CoA hydratase/isomerase family (change fold = 2.388), which is involved in fatty acid beta-oxidation (Müller-Newen, Janssen & Stoffel, 1995). The up-regulation of proteins associated with lipid metabolism, which can produce large amounts of energy, indicates that SYP-14288 cause a dramatic redistribution of metabolic processes associated with energy production. Indeed, it is interesting to note that this result demonstrates an increase in the production of acetyl-CoA associated with up-regulation of lipid metabolism, while the other findings of the current study (detailed above) suggest levels of acetyl-CoA associated with carbohydrate metabolism were reduced, which again indicates a compensatory mechanism in response to the putative uncoupling activity of SYP-14288.

Some of the proteins with altered expression were allocated to functional categories completely unrelated to energy metabolism. For example, several proteins belonging to the ABC Superfamily were found to be significantly up-regulated. Previous studies have shown that ABC transporters, which transfer various substrates across membranes, have been implicated in the detoxification of fungicides by excretion and the response to environmental stress (Van Den Brule et al., 2002; Jones & George, 2004). Given the strong evidence presented above that SYP-14288 act as an uncoupler of oxidative phosphorylation, it is possible that the up-regulation of ABC proteins could be an attempt to excrete SYP-14288 in order to maintain a proton gradient in the mitochondria of *Phytophthora capsici* and finally offset the toxicity of the fungicide. It was also found that elicitin, which is associated with the induction of hypersensitive cell death and other biochemical changes associated with the defense response of plants (Kamoun et al., 1993), was significantly up-regulated. This implies that signal transduction is an essential factor in the response of *Phytophthora capsici* to SYP-14288. The current study also found that an inorganic phosphate transporter was highly up-regulated. Given that phosphate enters the mitochondria via a phosphate transporter in exchange for OH^−^ or by co-transport with H^+^ (Karandashov et al., 2004), it is possible that the increased levels of this transporter could be another compensatory mechanism to address the disrupted proton gradient and reduced energy production resulting from SYP-14288 treatment. Several heat shock proteins, which are known to be involved in generalized stress responses (De Maio, 1999), were also found to be up-regulated. In this case it is possible the altered expression was induced in order to respond to the thermolytic requirements of *Phytophthora capsici* if its energy dissipative pathways were inhibited by SYP-14288. Although none of the proteins discussed in this section are directly linked to energy metabolism, they are consistent with indirect consequences resulting from the disruption of oxidative phosphorylation, and therefore provide further evidence that SYP-14288 has a similar mode of action as the uncoupler Fluazinam.

Some functional groups of protein were affected dramatically under SYP-14288 treatment. The pyrophosphatases, which could hydrolyse ATP and produce phosphate (Clair et al., 1997), may cause the activation of extra phosphate transfer. Acceleration of respiratory rate caused by activation of oxidoreductases could be a complementary mechanism of ATP shortage. And some ABC proteins and metal ion binding proteins could acted as transmembrane transporters to transfer protons crossing the inner membrane of mitochondria thus compensate for the disrupted proton gradient. All these functional groups may be the primary step of SYP-14288 function and then caused the downstream intricate biological reaction.

In summary, the results of the current study found extensive evidence that SYP-14288 has a wide ranging effect on the metabolism of *Phytophthora capsici* involving a multitude of different biochemical pathways. However, the study also indicated that the primary effect of SYP-14288 was a disruption of the proton gradient within the mitochondria by perturbing proton transporter or altering membrane permeability (Guo et al., 1991), and that most of the other changes were down stream responses to compensate for the reduced ATP of both the ATP synthetase and ATPase being significantly up-regulated, as well as other proteins associated with energy metabolism (especially those associated with respiration) in response to the altered proton motive force. This shift in energy metabolism resulted in the activation of compensatory mechanisms affecting carbohydrate and lipid metabolism as well as a reduction in amino acid biosynthesis. The study also found evidence that the up-regulation of transmembrane transporters and proteins associated with stress response might also cooperate to compensate for the disrupted proton gradient and other downstream effects. However, despite characterizing the metabolic pathways affected by SYP-14288 and possible target sites, the proteomic analysis failed to identify a specific target protein. Further investigation, including affinity chromatography assay, are therefore required to completely characterize the SYP-14288 mode of action in *Phytophthora capsici*. However, the proteomic data collected in the current study does provide important insight into the overall effect of SYP-14288 in *Phytophthora capsici*, which could be useful for the registration and application of this novel fungicide.

## Conclusion

The novel fungicide SYP-14288 has shown excellent activity against various plant pathogens including *Phytophthora capsici*, which is the causal agent of blight on hundreds of plants. The current study utilized iTRAQ to gain a greater understanding of the mode of action of SYP-14288. A total of 599 out of 1,443 proteins were considered to have significantly altered expression; most of these proteins were associated with carbohydrate metabolism, energy metabolism and their downstream biological functions, especially oxidoreductase activity and subsequent ATP production associated pathways. All results of the current study seem to indicate that SYP-14288 causes a shift in energy metabolism that results in the activation of compensatory mechanisms affecting carbohydrate and lipid metabolisms. All the data strongly implies that SYP-14288 works as an oxidative phosphorylation uncoupler. However, further investigation is still needed to completely characterize the mode of action of SYP-14288.

## Supplemental Information

10.7717/peerj.7626/supp-1 Supplemental Information 1 Primers for quantitative RT-PCR analysis.

10.7717/peerj.7626/supp-2 Supplemental Information 2 A total of 1,443 proteins were identified during the iTRAQ-LC-MS/MSanalysis of the two proteome libraries.

10.7717/peerj.7626/supp-3 Supplemental Information 3 The structural of SYP-14288 and Fluazinam.

10.7717/peerj.7626/supp-4 Supplemental Information 4 Molecular weight distribution of proteins identified by iTRAQ analysis of *P. capsici* isolate HD3 exposed to the novel fungicide SYP-14288.

10.7717/peerj.7626/supp-5 Supplemental Information 5 Raw data.

## Additional Information and Declarations

### Data Availability

The following information was supplied regarding data availability: The raw data are available in the Supplemental File.
