# Supplementary material for: Insights from the proteome profile of Phytophthora capsici in response to the novel fungicide SYP-14288
Source: PeerJ. 2019 Aug 27;7:e7626. doi: 10.7717/peerj.7626 (PMC6716503; doi:10.7717/peerj.7626)
Supplement: Supplemental Information 1 [file peerj-07-7626-s001.docx]

Supplemental Table S1

Primers for quantitative RT-PCR analysis

| Gene name | Primer | Primer sequence (5’-3’) |
| --- | --- | --- |
| ATP synthase gamma subunit | F | GAGGGCGACAACCTGTTC |
|  | R | GCTTACGGGTGGTCTTGG |
| ATP synthase F0 subunit 1 | F | TCGTTCCAACGTCGAGTTCG |
|  | R | TGAAAGCCTCAGCGATGGTC |
| ATP synthase beta subunit | F | GGACGGTCTCGTTCGTGG |
|  | R | GCTTGGCGTTGATGGGTC |
| putative ATPase | F | CGACTGTGGGCGTTGAGTTTGG |
|  | R | AGCCGATGAAGATGGCGATGAA |
| ATPase family AAA domain-containing protein 3A | F | CTGGCGTTCTCCATTGTTG |
|  | R | GTAAATTGCATTGGCTTCG |
| ATPase | F | GTTGAAGATGCGATTCCAGG |
|  | R | TGCTCACCGACTCAATAACC |
| ATP phosphoribosyltransferase | F | CATTATTGCGGAAAGCCAGAC |
|  | R | TCTCAAACTGCTTGAAATAGTCCC |
| cytochrome c oxidase subunit 2 | F | TCGGCATGTACGGCCTCAAGT |
|  | R | GCAGTCAGTGGCACGAGCAAT |
| long-chain-fatty-acid-CoA ligase | F | CGATCCTCCGCTACCTACTGA |
|  | R | GCGAGTGGGAGATACGAACAAT |
| acyl-CoA dehydrogenase, putative | F | AATACCTAATGGCTGAAA |
|  | R | TTATTTCTTGTACTCCTC |
| fumarate hydratase | F | GTGGGCACGGGACTCAATACG |
|  | R | CATGAGGGAGCAGGCGATGGT |
| malate dehydrogenase | F | TCGTGACCGACAAGGACTGGG |
|  | R | GCGGACATGGCACTGGAAAGC |
| triosephosphate isomerase | F | ACCAGAAGTTGCGGCAATGGA |
|  | R | CACAGAGCACTGCGGATGATAG |
| glucosylceramidase | F | CGAAGCTCAAGGCGGGTCAAG |
|  | R | AAGCCAAGCATGGTCTGGTACTGC |
| adenylosuccinate lyase | F | GAGAACATCTTGATGGCTTGCG |
|  | R | TCCACCTTGACACGCTTACCAG |
| aspartyl-tRNA synthetase | F | TTGTTGGTGGTAGCGTCCGTGAA |
|  | R | TTGTTGGTGGTAGCGTCCGTGAA |
| pleiotropic drug resistance protein | F | GGACCCGAAGAGCCCGAAAGA |
|  | R | CTTATCAGCACCCGACGACCC |
| ABC transporter-like protein | F | GATGCCGAACGAGAAGGTGG |
|  | R | TAGCTTGACGGGATGAGGT |
| elicitin | F | ACACCGAAACCCACGAAG |
|  | R | AGCCTTCTCAGCATGGTC |
| heat shock protein 90 | F | CCTCGCCGAGCTTCTCGA |
|  | R | GGTTATGTATTGCTCGTT |
| WS21 (40S ribosomal protein S3A) | F | GGAAAGAACAAACGCCTGAC |
|  | R | GTTGCGCTCCGAGAAGATA |
| UBC | F | GAAGCGGATCAACAAAGAGC |
|  | R | AAGCAGTGAGCAGATCGACA |
