# Supplementary figures and images for: Insights from the proteome profile of Phytophthora capsici in response to the novel fungicide SYP-14288
Source: PeerJ. 2019 Aug 27;7:e7626. doi: 10.7717/peerj.7626 (PMC6716503; doi:10.7717/peerj.7626)

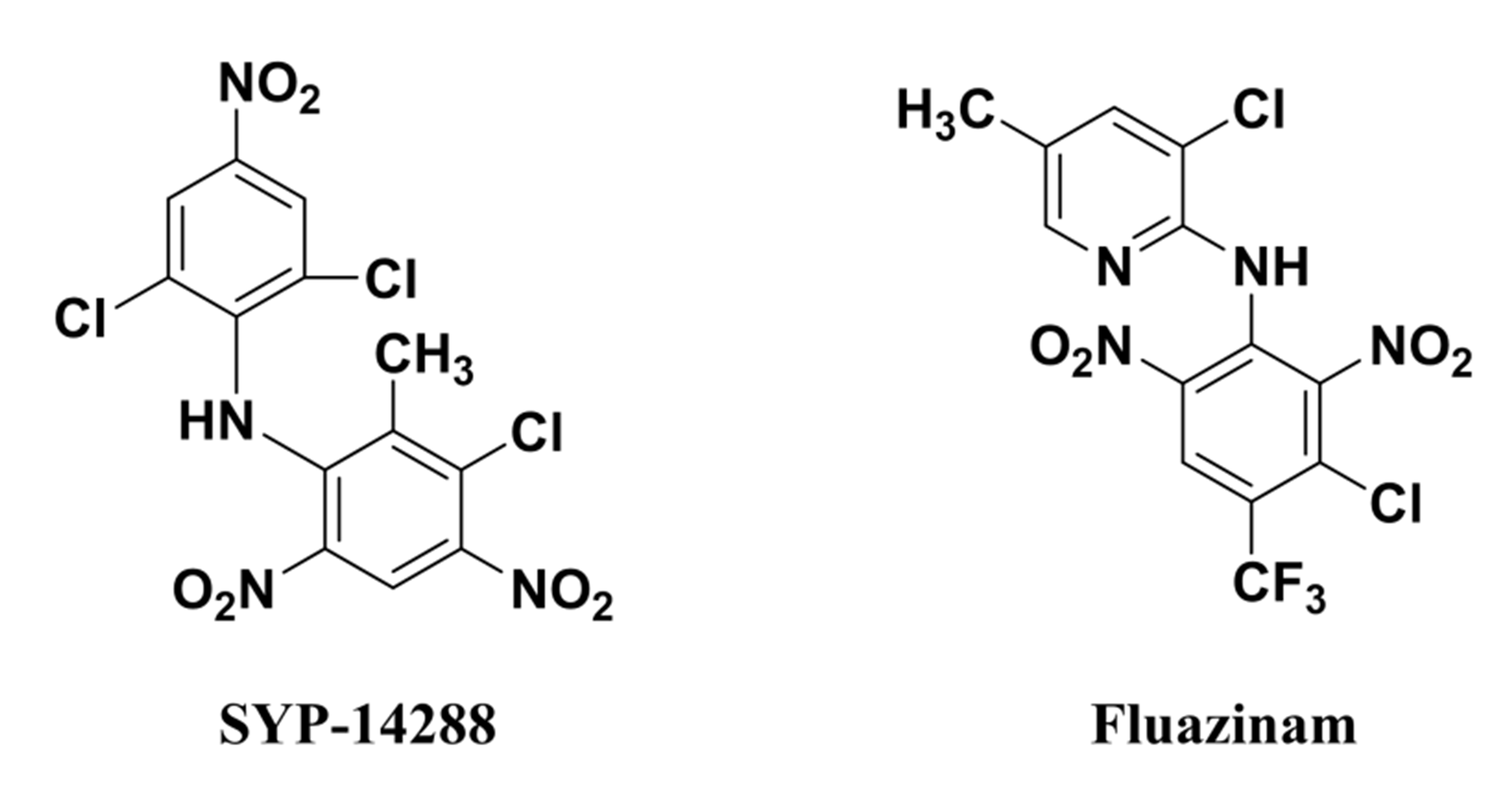

Supplement: Supplemental Information 3 [file peerj-07-7626-s003.png]

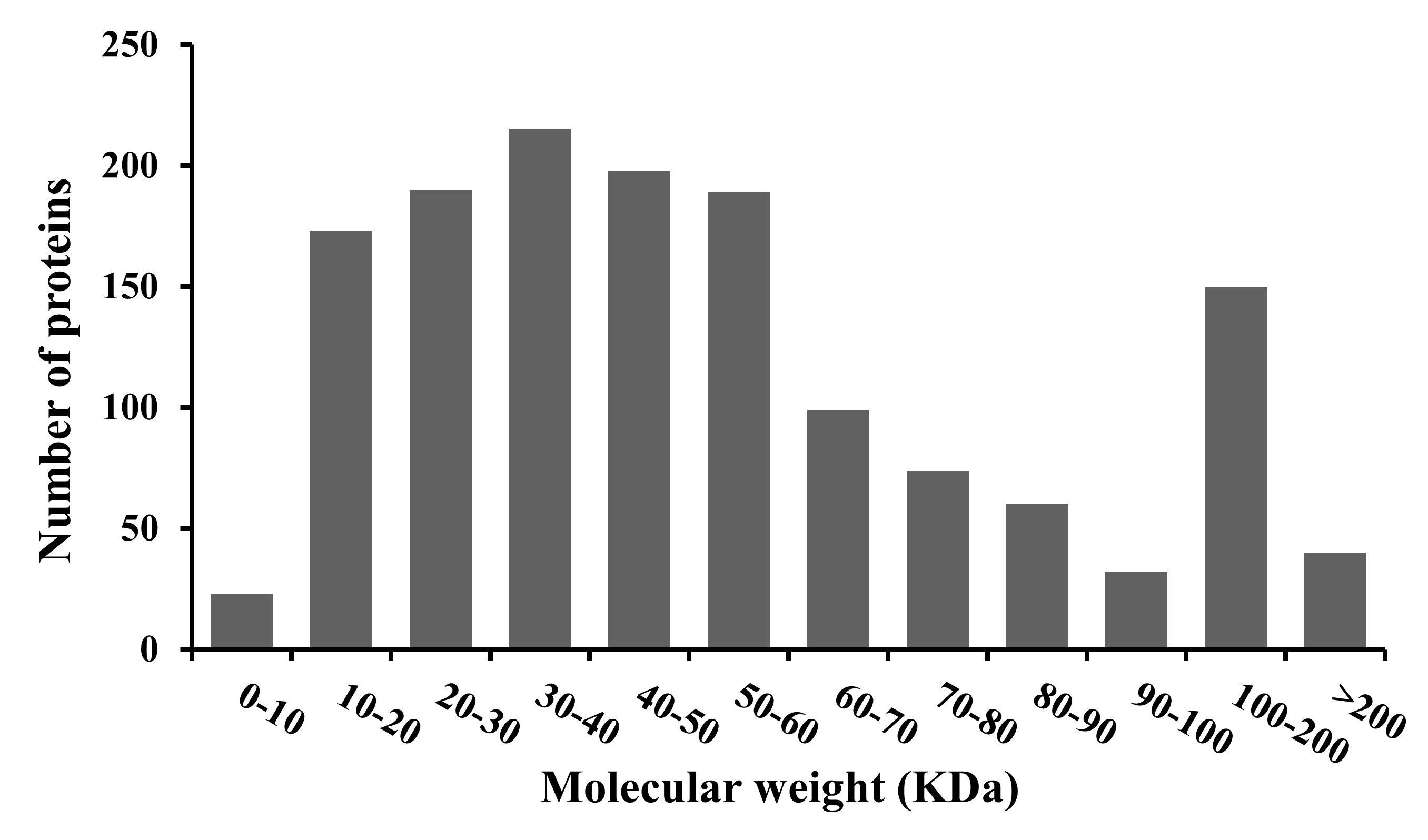

Supplement: Supplemental Information 4 [file peerj-07-7626-s004.png]
